# Protein S-palmitoylation modification: implications in tumor and tumor immune microenvironment

**DOI:** 10.3389/fimmu.2024.1337478

**Published:** 2024-02-13

**Authors:** Yijiao Chen, Yongsheng Li, Lei Wu

**Affiliations:** Department of Medical Oncology, Chongqing University Cancer Hospital, School of Medicine, Chongqing University, Chongqing, China

**Keywords:** protein S-palmitoylation, lipid modification, tumor cells, tumor immune microenvironment, immunotherapeutic strategies

## Abstract

Protein S-palmitoylation is a reversible post-translational lipid modification that involves the addition of a 16-carbon palmitoyl group to a protein cysteine residue via a thioester linkage. This modification plays a crucial role in the regulation protein localization, accumulation, secretion, stability, and function. Dysregulation of protein S-palmitoylation can disrupt cellular pathways and contribute to the development of various diseases, particularly cancers. Aberrant S-palmitoylation has been extensively studied and proven to be involved in tumor initiation and growth, metastasis, and apoptosis. In addition, emerging evidence suggests that protein S-palmitoylation may also have a potential role in immune modulation. Therefore, a comprehensive understanding of the regulatory mechanisms of S-palmitoylation in tumor cells and the tumor immune microenvironment is essential to improve our understanding of this process. In this review, we summarize the recent progress of S-palmitoylation in tumors and the tumor immune microenvironment, focusing on the S-palmitoylation modification of various proteins. Furthermore, we propose new ideas for immunotherapeutic strategies through S-palmitoylation intervention.

## 1 Introduction

S-palmitoylation is a highly conserved post-translational lipid modification of proteins that is widely present in eukaryotes. It plays a crucial role in various physiological processes by influencing protein structure, localization, transport, and function (1). Recent system-level analysis has revealed that S-palmitoylation affects more than 10% of the proteome, estimating that there are nearly 1,000 palmitoylated proteins in humans (2). Protein palmitoylation can be categorized into S-palmitoylation, N-palmitoylation, and O-palmitoylation based on the different acyl receptors (3). Among these modification, S-palmitoylation occurs on cysteine residue of a protein, involves a thioester linkage and is a reversible modification. N-palmitoylation can occur at both the N-terminus and the epsilon amino group of the protein via an amide bond. O-palmitoylation involves the attachment of palmitate to the serine and threonine residues of target protein via an ester bond (4). S-palmitoylated proteins are mainly membrane proteins, particularly transmembrane proteins and peripheral membrane proteins (5). The S-palmitoylation modification increases protein hydrophobicity and membrane binding ability, thereby altering protein structure, localization, transport, and function.

Protein S-palmitoylation plays an important role in tumor progression as many oncogenic proteins or tumor suppressors are palmitoylated (6). Recent studies have shown that protein S-palmitoylation in tumor cells or immune cells influences the tumor immune microenvironment by regulating the activation, depletion, and infiltration of immune cells (7, 8). The emergence of multiple immune checkpoint inhibitors and the success of various tumor immunotherapy clinical trials have greatly contributed to the advancement of tumor immunotherapy (9, 10). Understanding the impact of protein S-palmitoylation on the tumor immune microenvironment is a key area of research in this field. This review provides a comprehensive analysis of how S-palmitoylation regulates tumor cells and the tumor immune microenvironment. Furthermore, it explores potential ideas for more precise personalized treatment through the development of protein S-palmitoylation targeted drugs.

## 2 Dynamic regulation of protein S-palmitoylation

**Figure 1:**
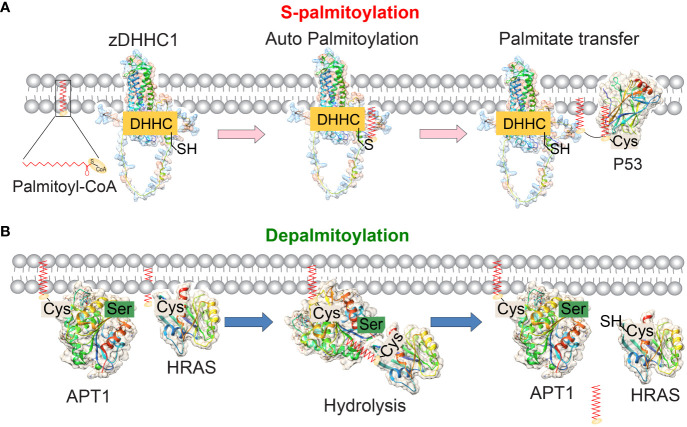
The reversible palmitoylation of proteins mediated by the enzymes zDHHC1 and APT1. **(A)** The molecular structures of human palmitoyltransferase zDHHC1 (predicted by SWISS-MODEL) and its substrate P53 (PDB ID: 1uol) are depicted. zDHHC-PATs are membrane proteins that contain 4 or 6 transmembrane domains. The enzyme’s catalytic DHHC motif is located in the cytoplasm. During the S-palmitoylation process, the DHHC domain of zDHHCs binds to palmitoyl-CoA and undergoes autopalmitoylation. Subsequently, the palmitate group is transferred to the cysteine residue of the substrate protein, thereby promoting the membrane localization of the substrates. **(B)** The molecular structures of human APT1 (PDB ID: 5sym) and its substrate protein HRAS (PDB ID: 1agp) are depicted. APT1 removes palmitic acid groups from the palmitoylated substrate. APT1/2 itself undergoes palmitoacylation and contains a hydrophobic pocket to accept the palmitoacylated substrates and hydrolyze them.

Unlike other permanent lipid attachments, S-palmitoylation can be reversed by cellular thioesterases, allowing for dynamic regulation of the local hydrophobicity of substrate proteins (11). Protein S-palmitoylation is catalyzed by palmitoyl S-acyltransferases (PATs) and acyl-protein thioesterases (APTs) enzymes (**Figure 1**) (4). Proteins can transition between palmitoylated and de-palmitoylated forms within timeframes ranging from seconds to hours (12). The dynamic process of S-palmitoylation can impact protein localization, accumulation, secretion, stability, and function by altering membrane affinity (13). Investigating how protein S-palmitoylation influences the function of specific proteins in both normal and cancer cells is a significant driving force behind current research in this field.

In humans, S-palmitoylation is catalyzed by a family of zinc finger and DHHC motif-containing palmitoyl acyltransferases (zDHHC-PATs). The zDHHC proteins have four to six transmembrane domains contain a signature Asp-His-His-Cys (DHHC) motif within the cysteine-rich domain in the intracellular loop (13, 14). The majority of zDHHC proteins are localized in the endoplasmic reticulum (ER) and Golgi apparatus, which are the primary sites of protein S-palmitoylation activity in mammalian cells (**Table 1**). The crystal structure analysis of human zDHHC20 has confirmed that the transfer of palmitoyl-CoA to substrate occurs in two steps (15, 16). zDHHC20 exhibits a teepee-like structure, wider at the cytoplasmic side and narrower at the membrane-inner side, with the DHHC active site positioned at the cytoplasmic junction between the transmembrane domains 2 and 3 (12). This position allows for interaction with both palmitoyl-CoA and substrate proteins at the membrane-cytosol interface. Palmitoyl-CoA first reacts with the cysteine residue in the DHHC motif, forming an acyl-intermediate and releasing free CoA-SH (12). Subsequently, this intermediate is transferred from the DHHC motif to the substrate proteins directly. Cysteine residues located near the catalytic DHHC motif coordinate two structural zinc atoms that are vital for the proper folding and function of the enzyme, but do not play a catalytic role in palmitic acid transfer (17). Despite sharing similar amino acid sequences, the 24 human zDHHC enzymes exhibit distinct abilities for self-acylation, indicating their diverse roles in tumor progression (18). It should be emphasized that although palmitate (C16:0) is the main lipid of endogenous S-acylated proteins, other fatty acids such as stearate (C18:0) and oleate (C18:1) can also be added to a protein cysteine residue (19). In addition, different cell types may exhibit different S-acylated lipid profiles, and in fact, the lipid profiles of S-acylated proteins in cells have been reported to be significantly influenced by the lipid composition of the extracellular environment (19).

**Table 1:** An overview of the function of zDHHCs in cancers.

Gene	Localization	Function	Target
**ZDHHC1**	ER, Golgi	zDHHC1 plays a crucial role in p53 palmitoylation at the C135, C176, and C275 residues, facilitating the nuclear translocation of p53. Additionally, it has a function in DNA virus-triggered and CGAS-mediated innate immune response, independent of its palmitoyltransferase activity. zDHHC1 acts as an activator of STING1 by promoting the cGAMP-induced oligomerization of STING1 and facilitating the recruitment of downstream signaling components.	P53, IFITM3
**ZDHHC2**	ER, Golgi	zDHHC2 functions as a mediator of AGK palmitoylation, facilitating AGK translocation to the plasma membrane and activating the PI3K-AKT-mTOR signaling pathway in RCC. It also plays a crucial role in cell adhesion by palmitoylating CD9 and CD151, thereby regulating their expression and function. Furthermore, zDHHC2 palmitoylates CKAP4, which in turn influences its localization to the plasma membrane. Additionally, zDHHC2 has the potential to palmitoylate LCK and regulate its localization to the plasma membrane.	AGK, LCK, CD9, CD151 CKAP4
**ZDHHC3**	Golgi	zDHHC3 is responsible for palmitoylating PPT1, leading to a reduction in PPT1 enzyme activity. It also plays a role in mediating APT2 palmitoylation at Cys2, which allows APT2 to stably bind to the membrane and search for potential substrates. Additionally, zDHHC3 palmitoylates ITGA6 and ITGB4, thereby regulating the localization, expression, and function of the alpha-6/beta-4 integrin in cell adhesion to laminin. Moreover, the palmitoylation of PD-L1 at Cys272 inhibits its ubiquitination-mediated lysosomal degradation. Lastly, zDHHC3 is involved in palmitoylating STING in the Golgi apparatus.	PPT1, APT2, ITGβ4, PD-L1, STING
**ZDHHC4**	ER, Golgi, Plasma membrane	zDHHC4 is involved in mediating the palmitoylation of GSK3β, which contributes to the promotion of tumorigenicity in temozolomide-resistant glioblastoma stem cells. This effect is achieved through the EZH2-STAT3 axis.	GSK3β
**ZDHHC5**	Plasma membrane	zDHHC5 participates in the uptake of fatty acids by palmitoylating CD36, facilitating its transportation to the plasma membrane. zDHHC5 is responsible for the palmitoylation of NOD1/2, enabling its localization to the membrane and subsequent induction of NF-κB signaling in response to peptidoglycan. Furthermore, ZDHHC5 plays a crucial role in regulating oligodendrocyte development by catalyzing STAT3 palmitoylation.	CTNND2, CD36, NOD1, NOD2, STAT3 and S1PR1
**ZDHHC6**	ER	zDHHC6 is responsible for palmitoylating ITPR1 in immune cells, which helps regulate the stability and function of ITPR1. Additionally, zDHHC6 palmitoylates MYD88, and when zDHHC6 is knocked down, it reduces MYD88 palmitoylation and TLR/MYD88 activation.	AEG-1, AMFR, calnexin, ITPR1 and TFRC
**ZDHHC7**	Golgi	zDHHC7 palmitoylates STAT3 at cysteine 108, facilitating its recruitment and phosphorylation at the cell membrane. Moreover, it plays a role in regulating the FAS signaling pathway by palmitoylating and stabilizing the receptor at the plasma membrane. zDHHC6 also palmitoylates SCRIB, controlling its localization to the plasma membrane, which indirectly affects cell polarity and differentiation. Furthermore, it palmitoylates JAM3, enhancing its expression at tight junctions and regulating its function in cell migration.	STAT3 FAS, SCRIB, ESR1, PGR and AR, GNAQ
**ZDHHC8**	Golgi	zDHHC8 is responsible for palmitoylating ABCA1, which plays a crucial role in regulating the localization of the transporter to the plasma membrane. This, in turn, affects the function of ABCA1 in cholesterol and phospholipid efflux. Additionally, zDHHC8 palmitoylates the D2 dopamine receptor DRD2, thereby regulating its stability and localization to the plasma membrane.	ABCA1, DRD2
**ZDHHC9/ZDHHC10**	ER, Golgi	zDHHC9/10 plays a crucial role in the palmitoylation of GLUT1 at C207, which enhances its localization to the plasma membrane and promotes a high level of glycolysis in glioblastoma. The zDHHC9-GOLGA7 complex serves as a specific palmitoyltransferase for HRAS and NRAS. Additionally, it may exhibit palmitoyltransferase activity towards the beta-2 adrenergic receptor/ADRB2, thereby regulating G protein-coupled receptor signaling.	GLUT1, HRAS, ADRB2
**ZDHHC11**	ER	zDHHC11 exhibits palmitoyltransferase activity towards NCDN and plays a role in regulating cell proliferation. It also has a function independent of its palmitoyltransferase activity, specifically in the DNA virus-triggered and CGAS-mediated innate immune response.	NCDN
**ZDHHC11B**	Membrane, Golgi	May play a role in cell proliferation.	–
**ZDHHC12**	ER, Golgi	zDHHC12 is responsible for mediating S-palmitoylation of CLDN3 at amino acid positions C181, C182, and C184. This modification contributes to the accurate localization and stabilization of CLDN3 on the plasma membrane, particularly in ovarian cancer. Additionally, zDHHC12 acts as an inhibitor of the NLRP3 inflammasome by facilitating the palmitoylation of NLRP3, ultimately promoting its degradation.	CLDN3, GPHN, NLRP3
**ZDHHC13**	ER, Golgi	zDHHC13-dependent Drp1 palmitoylation results in the normal occurrence of mitochondria fission-fusion processes. It acts as a palmitoyltransferase for HTT and GAD2.	Drp1, HTT MC1R, GAD2
**ZDHHC14**	ER, Golgi	zDHHC14 inhibits acute myeloid leukemia cellular differentiation. It also exhibits palmitoyltransferase activity towards the beta-2 adrenergic receptor/ADRB2, thereby regulating G protein-coupled receptor signaling. Additionally, it may play a role in cell differentiation and apoptosis.	ADRB2
**ZDHHC15**	Golgi	zDHHC15 palmitoylates IGF2R and SORT1, facilitating their localization to a specific subdomain of the endosomal membrane. This localization enables their interaction with the retromer cargo-selective complex. zDHHC15 also mediates STING palmitoylation in the Golgi apparatus and has the potential to catalyze the palmitoylation of GAP43.	IGF2R, SORT1, GAP43
**ZDHHC16**	ER	zDHHC16 is responsible for palmitoylating ZDHHC6, which affects the quaternary assembly, localization, stability, and function of ZDHHC6. Additionally, the palmitoylation of PCSK9 at cysteine 600 enhances the binding affinity between PCSK9 and PTEN.	ZDHHC6, PCSK9
**ZDHHC17**	Golgi	zDHHC17 palmitoylates Caspse6, preventing its dimerization and subsequent activation. Moreover, zDHHC17 may play a role in the sorting or targeting of crucial proteins involved in the initial stages of endocytosis at the plasma membrane.	CASP6, SNAP25
**ZDHHC18**	Golgi	Palmitoylation of MDH2, catalyzed by zDHHC18 at C138, results in the activation of mitochondrial respiration and promotes the malignancy of ovarian cancer. Additionally, it acts as a negative regulator of the cGAS-STING pathway by mediating the palmitoylation and inactivation of CGAS. zDHHC18 may also exhibit palmitoyltransferase activity towards the beta-2 adrenergic receptor/ADRB2, thereby regulating G protein-coupled receptor signaling.	MDH2, CGAS, HRAS, LCK, ADRB2
**ZDHHC19**	Cytoplasm, Golgi	Palmitoylation of SMAD3 by zDHHC19 promotes the activation of the transforming growth factor-beta (TGF-β) signaling pathway.	SMAD3, RRAS
**ZDHHC20**	Cytoplasm, Golgi, ER	zDHHC20 palmitoylates EGFR, leading to the activation of EGFR signaling. This, in turn, enhances cell migration and anchor-independent growth in lung cancer.	EGFR, IFITM3
**ZDHHC21**	Golgi, Plasma membrane	zDHHC21 exhibits specific catalytic activity in the palmitoylation of AK2, resulting in the activation of OXPHOS in leukemic blasts. Additionally, it palmitoylates FYN, thereby regulating its localization in hair follicles and playing a crucial role in epidermal homeostasis and hair follicle differentiation. The palmitoylation of PLCB1 and the subsequent regulation of its downstream signaling pathways may indirectly influence the function of the endothelial barrier and the adhesion of leukocytes to the endothelium. Furthermore, ZDHHC21 demonstrates palmitoyltransferase activity towards ADRA1D, positively modulating its activity and expression, potentially contributing to vascular contraction. It may also be involved in the palmitoylation of eNOS and LCK.	AK2, EGFR, eNOS, LCK, AR, FYN, ADRA1DP, PLCB1
**ZDHHC22**	ER, Golgi	zDHHC22 palmitoylates mTOR, leading to reduced mTOR stability and decreased activation of the AKT signaling pathway. Additionally, zDHHC22 palmitoylates KCNMA1, thereby regulating its localization to the plasma membrane.	mTOR, KCNMA1, CNN3
**ZDHHC23**	Golgi	zDHHC23 palmitoylates KCNMA1, playing a role in its localization to the plasma membrane.	KCNMA1
**ZDHHC24**	Membrane	–	–

Since there is no consistent S-palmitoylation sequence, how the zDHHC enzyme selects specific substrate proteins for modification is not fully understood. S-palmitoylation proteins can usually be catalyzed by more than one zDHHC enzyme, but a specific zDHHC enzyme is usually more potent than other enzymes for the substrate palmitoylation in cells (18). Interestingly, certain zDHHC enzymes have unique substrate binding preferences. For some proteins, proteins palmitoylation requires other lipidation events to occur on amino acid residues close to palmitoylated Cys residues (e.g., RAS farnesylation, SRC family myristoylation), and these modifications help substrate locate to zDHHC-enriched membrane region (20). zDHHC13 and zDHHC17 contain unique C-terminal ankyloprotein repeat domains that bind certain substrate and enhance their membrane localization, promoting substrate S-palmitoylation by other zDHHC enzymes (18). In addition, the palmitoylation process of some zDHHC enzymes such as zDHHC9 and zDHHC6 requires the involvement of a helper protein such as Selenoprotein K (21). Therefore, these helper proteins may contribute to substrate selection. For some protein substrates, only certain portions of cysteine residues can be S-palmitoacylated. Progress has been made in computational prediction, but experiments are still needed to determine which specific cysteines in proteins will be modified. It was found that S-palmitoylation of membrane proteins preferentially occurs at cysteine located in the 8 angstroms range of the membrane-cytoplasmic interface, which provides a structural constraint on the S-palmitoylation potential of certain residues of the substrate protein (22). These results suggest that further studies are needed to determine how substrate conformation or binding affects protein S-palmitoylation.

Depalmitoylation modification in mammalian cells is catalyzed by APTs and palmitoyl-protein thioesterases (PPTs), as well as the mammalian α/β hydrolase domain-containing proteins (ABHDs) (**Table 2**) (23). Soluble APT2 weakly interacts with the cell membrane through its β tongue. It can separate from the membrane or encounter zDHHC3/7 (24). In this case, zDHHC3/7 mediates APT2 S-palmitoylation at Cys2, allowing APT2 to stably bind to the membrane and search for potential substrates (25). When APT2 encounters a substrate, it triggers the extraction of an acyl chain from the membrane, causing it to move into the APT2 hydrophobic pocket. With this ideal positioning, APT2 hydrolyzes the substrates (26). Interestingly, inhibiting APT1 expression or its thioesterase activity markedly increases the membrane localization of APT2, while shRNA suppression of APT2 has no effect on the membrane localization of APT1 (27). These results indicate that APT1 and APT2 S-palmitoylation link cytosol-membrane trafficking of their substrates, facilitating their membrane localization and function. PPT1 plays a critical role in protein depalmitoylation. However, the substrates of PPT1 and the catalytic process are largely unknown. A recent study used a 2-step proteomic approach to identify 138 novel PPT1 substrates (28). They found that the validated substrates of PPT1 frequently catalyze cysteine residues that participate in disulfide bonds. The depalmitoylated cysteines catalyzed by PPT1 spontaneously form disulfide bonds in the oxidative environment, suggesting a novel function of PPT1 in oxidative stress. ABHD17A, ABHD17B, and ABHD17C are novel depalmitoylation enzymes that have been recently identified as regulators of N-Ras, Psd-95, and MAP6 depalmitoylation (29, 30). N-Ras depalmitoylation by ABHD17 is required for its re-localization to internal cellular membranes (31).

**Table 2:** The function of depalmitoylation enzymes in cancers.

Gene	Localization	Function	Target
**APT1**	Membrane, Cytoplasm, ER, Nucleus	APT1 and APT2 are enzymes involved in the hydrolysis of fatty acids from S-acylated cysteine residues in proteins. APT1 specifically acts on proteins such as trimeric G alpha proteins, HRAS, and ADRB2, displaying depalmitoylating activity.	HRAS NRAS, KCNMA1, ADRB2, CKAP4, LPR6
**APT2**	Cytoplasm	APT2 depalmitoylates phosphorylated STAT3, facilitating its translocation to the nucleus. Additionally, APT2 also hydrolyzes fatty acids from S-acylated cysteine residues in proteins like GAP43, ZDHHC6, and trimeric G alpha proteins.	STAT3, GAP43, ZDHHC6, HRAS
**PPT1**	Lysosome, Secreted	PPT1 is responsible for removing thioester-linked fatty acyl groups, such as palmitate, from modified cysteine residues in proteins or peptides during lysosomal degradation. It has a preference for acyl chain lengths of 14 to 18 carbons. PPT1 frequently catalyzes cysteine residues that participate in disulfide bonds.	–
**PPT2**	Lysosome	PPT2 exhibits the highest S-thioesterase activity for the acyl groups palmitic and myristic acid, followed by other short- and long-chain acyl substrates. However, PPT2 is unable to remove palmitate from peptides or proteins.	–
**ABHD17A**	Cell membrane	ABHD17A exhibits depalmitoylating activity towards NRAS and DLG4/PSD95, and it may also have depalmitoylating activity towards MAP6.	MAP6, NRAS
**ABHD17B**	Cell membrane	ABHD17B demonstrates depalmitoylating activity towards DLG4/PSD95 and GAP43.	DLG4/PSD95, GAP43
**ABHD17C**	Cell membrane	ABHD17C hydrolyzes fatty acids from S-acylated cysteine residues in proteins, and it also exhibits depalmitoylating activity towards NRAS and DLG4/PSD95.	NRAS, DLG4/PSD95

Palmitoyl proteins play a crucial role in complex regulatory networks. For instance, zDHHC5, zDHHC6, and zDHHC8 are responsible for catalyzing the S-palmitoylation of cysteine residues that are located outside the DHHC motif. These modifications are essential for ensuring the proper localization and functioning of the enzyme (12). Recent research has demonstrated that zDHHC16 physically interacts with zDHHC6 and palmitoylates the Cys328, Cys329, and Cys343 residues of the C-terminal Src Homology 3 (SH3) domain of zDHHC6 (32). The S-palmitoylation of zDHHC6 by zDHHC16 enhances the overall stability and activity of zDHHC6. However, it is important to note that different configurations of modifications yield varying activity and stability profiles. The S-palmitoylation of zDHHC6 on these C-terminal cysteine residues is reversible through APT2, with the conversion occurring particularly rapidly at Cys328. The thioesterases belonging to the APT1/2 and ABHD families are also palmitoylated, and this modification is crucial for their proper localization and functioning (25). PPT1 is another protein that undergoes S-palmitoylation by zDHHC3 and zDHHC7, although in this case, S-palmitoylation reduces enzyme activity. Moreover, both the PAT enzymes (*e.g.*, zDHHC3, zDHHC13) and the APT enzymes (*e.g.*, APT1, APT2) can be regulated through phosphorylation or ubiquitination in cancer and other cells, further adding to the complexity of this regulatory network. Thus, a complex web of interconnected modifications regulates the localization and functioning of both zDHHC and thioesterase proteins, which has significant implications for the regulation of cancer-associated proteins.

## 3 Proteins S-palmitoylation in cancer

Protein S-palmitoylation plays a crucial role in cancer initiation, cancer cell growth, survival, and modulation of the anti-tumor immune response. Aberrant S-palmitoylation of proteins may indicate functional alterations in cancer. Several studies have demonstrated that the zDHHC family members can both inhibit and promote tumorigenicity by regulating S-palmitoylation of important proteins, including epidermal growth factor receptor (EGFR), Ras, and PD-L1, etc. (33, 34).

### 3.1 Protein S-palmitoylation in the regulation of oncogenic or tumor-suppressive signaling

**Figure 2:**
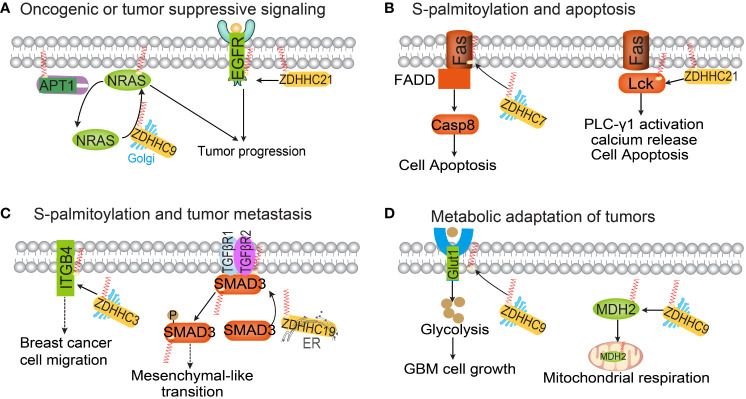
The functional roles of protein S-palmitoylation in cancer. Palmitoylation regulates oncogenic or tumor-suppressive signaling pathways **(A)**, apoptosis **(B)**, tumor metastasis **(C)** and tumor metabolism **(D)**.

Numerous carcinogenic or tumor-suppressive proteins involved in tumorigenesis, metastasis, or drug resistance are regulated by S-palmitoylation. Studies have shown that zDHHC9-mediated S-palmitoylation of oncogenic NRAS is essential for its localization on the plasma membranes and downstream signaling activation, promoting the occurrence of leukemia (34, 35). APT1 has been found to depalmitoylate H-Ras and N-Ras, reducing their affinity to the plasma membrane and releasing them from it, allowing diffusion back into the Golgi apparatus (36, 37). Therefore, the S-palmitoylation-depalmitoylation cycle of N-Ras and H-Ras regulates the transport of Ras between the Golgi apparatus and the plasma membrane (**Figure 2**). EGFR, a growth factor receptor critical for cell proliferation and differentiation (38), S-palmitoylation of EGFR by zDHHC1, zDHHC2, and/or zDHHC21 on Cys797 is essential for the stability, membrane localization, dimerization, and activation of this receptor and increases cell migration and anchor-independent growth in lung cancer (39). Fatty acid synthase (FASN), a major lipogenic enzyme that synthesizes palmitic acid (40), induces an oncogenic phenotype when overexpressed in normal cells. This transformation involves enhanced lipid synthesis and an increase in phosphorylation and expression of EGFR (41). EGFR activation is involved in FASN-dependent palmitoylation, which is required for both ligand-dependent and ligand-independent activation of EGFR and occurs within the cell. Treatment of prostate and lung cancer cells with the FASN inhibitor cerulenin or the palmitoylation inhibitor 2-bromopalmitate (2-BP) reduces EGFR levels on the plasma membrane while increasing lysosome levels (42). A recent published study also reported the lipid-rich microenvironment activates palmitate biosynthesis in metastatic colorectal cancer cells by up-regulating FASN. The increased intracellular palmitate promotes EGFR palmitoylation, thereby activating EGFR signaling. The FASN inhibitor orlistat inhibits EGFR palmitoylation and inhibits the stem cell properties of colorectal cancer (CRC) cells (43). EGF stimulates the S-palmitoylation of the oncogenic protein CDCP1, thereby blocking the degradation of CDCP1 (44). This study establishes a connection between S-palmitoylation events and EGFR signaling. However, further research is required to fully understand the specific mechanisms involved.

The membrane protein claudin-3 (CLDN3) is crucial for the formation and maintenance of tight junctions and is highly expressed in various cancers (45). In ovarian cancer, zDHHC12 mediates S-palmitoylation of CLDN3 at C181, C182, and C184, which contributes to its proper localization and stabilization on the plasma membrane. zDHHC22 is significantly upregulated in renal cancer cells (RCC) resistant to tyrosine kinase inhibitors (46). In RCC, zDHHC2 mediates AGK S-palmitoylation, facilitating AGK translocation to the plasma membrane and activating the PI3K-AKT-mTOR signaling pathway (47). These findings indicate the existence of a zDHHC2-AGK signaling axis and suggest that targeting zDHHC2 could enhance the effectiveness of sunitinib in RCC treatment (47). zDHHC22 expression is significantly reduced in estrogen receptor-negative breast cancer tissues, and its expression is positively correlated with the clinical prognosis of breast cancer patients (46). Through S-palmitoylation, zDHHC22 decreases mTOR stability and suppresses AKT signaling pathway activation. Restoring zDHHC22 expression sensitizes tamoxifen-resistant MCF-7 cells to tamoxifen treatment (46). Proprotein convertase subtilisin/kexin type 9 (PCSK9) plays a crucial role in cholesterol metabolism and antitumor immune responses (48). Abnormal upregulation of PCSK9 promotes cell proliferation and sorafenib resistance in HCC. zDHHC16-mediated S-palmitoylation of PCSK9 at cysteine 600 enhances the binding affinity between PCSK9 and tensin homolog (PTEN) (49). S-palmitoylation of PCSK9 leads to lysosome-mediated PTEN degradation and subsequent AKT activation (50). Inhibiting PCSK9 S-palmitoylation suppresses AKT phosphorylation and enhances the antitumor effects of sorafenib (49).

P53 is a well-known tumor suppressor that regulates cell proliferation and apoptosis. The S-palmitoylation of p53 at the C135, C176, and C275 residues, mediated by zDHHC1, has been reported to play necessary roles in p53 nuclear trafficking and subsequent pathway activation (**Figure 1A**) (51). Conversely, p53 epigenetically regulates zDHHC1 expression by recruiting DNMT3A and HDAC1 to the promoter region (51). This regulatory feedback loop could help explain the function of zDHHC family members and may be utilized for targeted therapy.

### 3.2 S-palmitoylation and apoptosis

Apoptosis, a cell-intrinsic mechanism for suicide, plays a pivotal role in inflammatory diseases and tumors (52). Recent studies have identified dysregulated S-palmitoylation of apoptosis-related proteins in many human cancers. For instance, the TNF family receptor Fas can undergo S-palmitoylation, as revealed by metabolic labeling of [3H] palmitate in HEK293 cells (53, 54). zDHHC7-mediated S-palmitoylation of Fas leads to an increase in the active form of caspase-8 (55). Decreased expression of zDHHC7 inhibits the expression of Fas in the plasma membrane of SW480 cells, thereby suppressing FASL-induced apoptosis. While Fas is located on various cell surfaces, Fas ligand (FasL) is primarily found on activated T cells and natural killer (NK) cells (56). Dysregulation of Fas/FasL signaling compromises immune function. FasL can be palmitoylated at residue cysteine 82, and mutants at this site show significantly lower cell death compared to controls (57). S-palmitoylation of the Fas signaling member Lck is essential for Fas-mediated apoptosis (58). The binding of the Fas receptor to its ligand Fas results in a rapid and substantial increase in the S-palmitoylation level of the tyrosine kinase Lck. Inhibition of Lck S-palmitoylation not only disrupts proximal Fas signal transduction events but also confers resistance to Fas-mediated apoptosis. zDHHC21, a palmitoyltransferase, functions as a PAT that controls Lck activation. Knockout of zDHHC21 eliminates the activation and downstream signaling of Lck following Fas receptor stimulation (59).

Death receptor-6 (DR6) is a newly discovered member of the Death receptor subfamily that can induce apoptosis and/or activate NF-κB and JNK/SAPK family stress kinases in specific cell types (60). The S-palmitoylation of DR6 occurs at Cys368, but the relationship between DR6 S-palmitoylation and apoptosis requires further investigation. In COS7 cells, the increase in caspase-6 S-palmitoylation at the C264 and C277 sites, mediated by zDHHC17, leads to a decrease in caspase-6 activation (61). Additionally, S-palmitoylation can also occur in Bcl-2 family proapoptotic proteins. S-S-palmitoylation of BAX at Cys-126 is crucial for the apoptosis process as it affects its localization to the mitochondria (62). The mutation C126 significantly reduces the number of apoptotic cells and the activation of caspase-3. These findings collectively indicate that S-palmitoylation plays an essential role in the apoptosis process of immune cells and tumor cells.

### 3.3 S-palmitoylation and tumor metastasis

Aberrant S-palmitoylation has been shown to contribute to tumor initiation and growth, and its potential role in tumor metastasis has been investigated. Tetraspanins, which are small membrane proteins, have been found to promote the adhesion and metastasis of tumor cells (63). The S-palmitoylation of integrin subunits beta-4 (ITGB4), alpha-3 (ITGA3), and alpha-6 (ITGA6) has an impact on the formation of integrin tetraspanin complexes (64). Additionally, ITGA6 and ITGB4 collaborate with EGFR and ERBB2 to promote various aspects of tumor progression and metastasis (65). It is crucial to conduct studies to determine how different S-palmitoylation of proteins interferes with EGF receptor-integrin dynamics within tetraspanin complexes. Ras-related protein RAB27A is significantly overexpressed in oral squamous cell carcinoma (66). RAB27A regulates the S-palmitoylation of EGFR via zDHHC13 in oral squamous cell carcinoma (66). The laminin-binding integrin α6β4 plays a key role during tumor cell metastasis (67).

Several studies have indicated that the S-palmitoylation of α6β4 protein by zDHHC3 inhibits the degradation of α6β4. This inhibition is likely due to a decrease in endosomal exposure to cathepsin D (68). Flotillin-1 is a membrane-associated protein that plays a role in multi-cellular signaling events in cells (69). It has been observed that Flotillin-1 is overexpressed in numerous solid tumors, and its S-palmitoylation contributes to its stability and subsequent metastatic capabilities in breast cancer cells and experimental metastasis models (70, 71). Therefore, targeting flotillin-1 S-palmitoylation could be a promising approach for addressing breast cancer metastasis. MUC1, a mucin-like protein located on the apical membrane of epithelial cells, is also associated with tumor metastasis (72). S-palmitoylation of MUC1 modulates its recycling from endosomes to the plasma membrane (73), and the intracellular localization of MUC1 is altered during breast cancer metastasis (74). CCR5 is a CC chemokine receptor expressed on immune cells such as memory lymphocytes, macrophages, and dendritic cells. Weinberg and colleagues have discovered that CCR5 undergoes S-palmitoylation at three cysteine residues within its C-terminal region (75). Palmitoylated cysteines are known to have a significant role in the intracellular trafficking of CCR5. When CCR5 S-palmitoylation is eliminated, it reduces the surface expression of CCR5 by trapping the receptor in organelles, leading to its degradation. This is particularly relevant in breast cancer metastasis to bone, as CCR5 S-palmitoylation facilitates tumor metastatic spread through CCL5-CCR5 chemokine signaling. Another important factor in tumor metastasis is SMAD3, which is involved in the activation of the transforming growth factor-beta (TGF-β) signaling pathway (76). S-palmitoylation of SMAD3, mediated by palmitoyltransferase zDHHC19, promotes its activation. Furthermore, the interaction between SMAD3 and EP300 promotes the expression of mesenchymal markers in the mesenchymal subtype of glioblastoma multiforme (GBM). Therefore, targeting Smad3 S-palmitoylation could be a crucial molecular approach in combating tumor metastasis (77).

### 3.4 Protein S-palmitoylation in the metabolic adaptation of tumors

The relationship between cellular metabolic reprogramming and protein S-palmitoylation is inherently connected, as S-palmitoylation involves the covalent attachment of palmitic acid to proteins. In cancer cells, the addition of the saturated fatty acid palmitate enhances the synthesis of palmitoyl-CoA, thereby upregulating protein S-palmitoylation (78). This direct involvement of central metabolites in post-translational modifications allows tumor cells to integrate information from the microenvironment and effectively regulate cellular processes. Consequently, these palmitoylated metabolic proteins are believed to influence cancer cell metabolism. For instance, glucose transporter (GLUT1), a transmembrane protein responsible for glucose uptake, is frequently upregulated in various cancer types (79). The localization of GLUT1 to the plasma membrane is crucial for its function in glucose uptake. Studies have shown that zDHHC9 mediates the S-palmitoylation of GLUT1 at C207, promoting its plasma membrane localization and resulting in heightened glycolysis in glioblastoma (80). Malate Dehydrogenase (MDH2), a key enzyme in the TCA cycle, catalyzes the reversible conversion of malate to oxaloacetate. S-palmitoylation of MDH2 by zDHHC18 at C138 leads to increased MDH2 activity (81). This S-palmitoylation event activates mitochondrial respiration and promotes the malignancy of ovarian cancer. Additionally, zDHHC21 has recently been identified as a critical regulator of oxidative phosphorylation activation in acute myeloid leukemia cells. zDHHC21 specifically catalyzes the S-palmitoylation of mitochondrial adenylate kinase 2 (AK2) and activates OXPHOS in these leukemia cells (82). Depletion of ZDHHC21 induces the differentiation of acute myeloid leukemia cells and weakens their stemness potential (83).

Activated lipid metabolism has been found to promote cancer development both *in vitro* and *in vivo* (11, 84). Treatment with exogenous palmitate enhances Src-dependent mitochondrial β-oxidation, increases the level of Src kinase localized in the cell membrane, and activates Src-mediated downstream MAPK and FAK signaling (78). These results reveal that dietary palmitate, in collaboration with elevated Src kinase, accelerates prostate tumor progression (78). CD36, a fatty acid transporter, is widely expressed in immune cells and tumor cells (85). CD36 can be palmitoylated by zDHHC4 and zDHHC5 at both N-terminal and C-terminal cysteine residues (86). zDHHC4 and zDHHC5 function at different subcellular localizations and regulate CD36 S-palmitoylation, targeting it to the plasma membrane for fatty acid uptake (87). Inhibiting CD36 S-palmitoylation reduces the hydrophobicity of the CD36 protein and its localization in the plasma membrane of hepatocytes. Depletion of either zDHHC4 or zDHHC5 disrupts the capability of adipose tissues to uptake fatty acids (88). These findings demonstrate the critical role of zDHHC4 and zDHHC5 in regulating CD36 S-palmitoylation and fatty acid uptake.

## 4 Proteins S-palmitoylation in anti-tumor immunity

**Figure 3:**
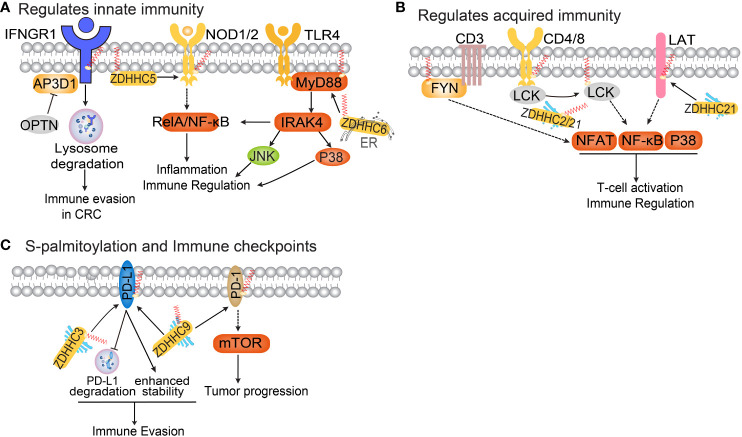
The functional roles of protein S-palmitoylation in anti-tumor immunity. **(A)** Palmitoylation regulates the IFNGR, NOD and TLR pathways. **(B)** Protein S-palmitoylation in T cell activation and immune regulation. **(C)** Protein S-palmitoylation regulates immune checkpoints.

Numerous palmitoylated proteins play a crucial role in IFN-γ, Nucleotide oligomerization domain (NOD)-like receptors 1 and 2 (NOD1/2), Stimulator of interferon genes (STING), and JAK-STAT signaling pathways, which are closely associated with anti-tumor immunity (3, 89). Recent studies have also started investigating the impact of protein S-palmitoylation on the regulation of the tumor immune microenvironment, particularly in relation to IFN-γ and PD-1/PD-L1 signaling (**Figure 3**) (33). Understanding the influence of protein S-palmitoylation on the tumor immune microenvironment is a significant driving force behind current research in this field.

### 4.1 S-palmitoylation regulates innate immunity

IFN-γ is a cytokine secreted by various activated immune cells, including NK and CD8^+^ T cells (90). Binding of IFNγ to its receptor activates the JAK-STAT pathway, inducing the expression of classical interferon-stimulated genes that play a critical role in antitumor responses (90). Recent research has discovered that S-palmitoylation of IFN-γ receptor 1 (IFNGR1) at cysteine 122 acts as a sorting signal for IFNGR1 lysosomal degradation, mediated by AP3D1 in CRC (91, 92). OPTN, which is lost in early-stage human CRC, interacts with AP3D1 to hinder its recognition of IFNGR1, thereby maintaining IFNGR1 stability and the integrity of downstream MHC-I signaling. This promotes antigen presentation to T cells and inhibits CRC progression (92).

NOD1/2 are intracellular pattern-recognition proteins that recognize peptidoglycan associated with microorganisms. The activation of NOD signaling is crucial for host defense against infections (93). Studies have demonstrated that zDHHC5 palmitoylates NOD1/2, which is necessary for its membrane localization and induction of NF-κB signaling in response to peptidoglycan (94). Toll-like receptor 4 (TLR4) is a pattern recognition receptor expressed by various innate immune cells, including macrophages, dendritic cells, and neutrophils (95). TLR4 participates in inflammasome activation by triggering downstream myeloid differentiation primary response protein (MYD88) signaling, leading to the release of inflammatory factors TNF-α, IL-1β, and IL-18 (96). The binding of IRAK4 to the MYD88 intermediate domain and subsequent signal activation require MYD88 S-palmitoylation at cysteine 113 (97). The S-palmitoylation of MYD88 is facilitated by *de novo* fatty acid synthesis and CD36-mediated incorporation of exogenous fatty acids. zDHHC6 is responsible for palmitoylating MYD88, and knockdown of zDHHC6 reduces MYD88 S-palmitoylation and impairs TLR/MYD88 activation upon lipopolysaccharide stimulus in neutrophils (97).

STING plays a crucial role in the innate immune response during infection (98). Research has shown that STING expression is elevated in patients with RCC and controls tumor growth through non-standard innate immune signaling involving the maintenance of mitochondrial ROS and calcium homeostasis (99). STING interacts with mitochondrial voltage-dependent anion channel (VDAC2) via STING-C88/C91 palmitoylation. zDHHC3, zDHHC7, and zDHHC15 palmitoylate STING in the Golgi apparatus of C89/91 (99). Depletion of STING enhances the formation of mitochondria-ER contacts mediated by VDAC2/grp75, leading to increased levels of mitochondrial ROS/calcium and damage to mitochondrial function. Recently, covalently bound small molecule inhibitors that target STING palmitoylation have been developed, further confirming the critical role of STING palmitoylation in its assembly into polymeric complexes in the Golgi apparatus and the activation of downstream STING-triggered inflammatory signals (100). These studies reveal the significance of S-palmitoylation of STING in regulating mitochondrial function and growth in RCC, providing a rationale for targeting STING palmitoylation in the treatment of RCC.

### 4.2 S-palmitoylation regulates acquired immunity

T cells play a crucial role in the adaptive immune response. Recent evaluation of the palmitome in primary T cells and Jurkat T cells revealed a pool of 120 palmitoylated proteins (101). This pool includes both well-known palmitoylated proteins and 92 previously unidentified palmitoylated proteins *in vivo*. Among the known palmitoylated proteins are ERBB2IP, FASN, CD44, and products of 52 genes associated with breast cancer metastasis to bone, lung, or brain (102). The study also demonstrated that certain PAT enzymes are themselves palmitoylated, suggesting the presence of a feedback mechanism. Furthermore, differential S-palmitoylation of T-cell surface antigens (mono- versus dual-lipidation) was observed, which likely contributes to increased functional flexibility. Previous studies have shown that S-palmitoylation regulates various components of the T-cell receptor (TCR) signaling pathway (59). S-palmitoylation has also been found to impact the T-cell co-receptors CD4 and CD8, as well as associated signaling molecules such as SRC family kinases LCK and FYN (103). Specifically, S-palmitoylation of CD4 on cysteine residues Cys396 and Cys399 near the membrane is associated with LCK, and together they enhance the enrichment and localization of CD4 in lipid rafts (3). Similarly, S-palmitoylation of CD8b is essential for the effective functioning of the CD8 co-receptor, as it increases the association of CD8 with p56lck and enhances p56lck activation in lipid rafts (104). In addition to N-terminal glycine myristoylation, LCK is palmitoylated on Cys3 and Cys5 (105). Both zDHHC2 and zDHHC21 have been identified as enzymes responsible for palmitoylating LCK (89, 106). S-palmitoylation plays a critical role in the plasma membrane targeting of LCK and the phosphorylation of its substrates, such as CD8. Knockdown of zDHHC21 leads to decreased LCK S-palmitoylation and subsequently reduced T-cell activation.

Calcium signaling plays a crucial role in T cell signaling, with calcium increases primarily mediated by 1-phosphatidylinositol 4,5-bisphosphate phosphodiesterase gamma-1 (PLC-γ1). PLC-γ1 activates the production of inositol triphosphate, which then binds to the inositol triphosphate receptor, triggering an increase in calcium levels within the cell membrane (107). zDHHC21 palmitoylates PLC-γ1, and its activity is dynamically regulated by TCR signaling (59). Furthermore, the T cell activation adapter LAT undergoes S-palmitoylation at Cys26 and Cys29. This S-palmitoylation helps LAT efficiently distribute to membrane microdomains and enables its tyrosine phosphorylation. The plasma membrane targeting function of S-palmitoylation is crucial in TCR signaling, and the absence of LAT S-palmitoylation results in T cell inactivity (108).

JAK2/STAT3 is a crucial signaling pathway that plays a significant role in promoting inflammation and tumor progression (109). The activation of JAK2 occurs when extracellular IL-6 binds to its receptor on the cell membrane, subsequently leading to the activation of STAT3 signaling. Once phosphorylated, STAT3 translocates to the nucleus and functions as a transcription factor, facilitating the expression of downstream genes. In this context, zDHHC7 plays a noteworthy role by palmitoylating STAT3 at cysteine 108, which enhances its membrane recruitment and phosphorylation (110). Interestingly, phosphorylated STAT3 is depalmitoylated by APT2, allowing it to translocate to the nucleus. This palmitoylation-depalmitoylation cycle augments STAT3 activation, thereby promoting Th17 cell differentiation (110). The excessive activation of TH17 cells has been linked to various inflammatory diseases, including inflammatory bowel disease (IBD). In a mouse model, the inhibition of APT2 through pharmacological means or the knockout of zDHHC7 has been found to alleviate the symptoms associated with IBD (110). These findings not only propose a potential therapeutic strategy for treating IBD but also provide insights into the role of S-palmitoylation in regulating cell signaling, which could have broader implications for understanding the functional significance of various S-palmitoylation events.

### 4.3 S-palmitoylation regulates immune checkpoints

Immune checkpoint inhibitors have been widely utilized in tumor immunotherapy. PD-L1, a transmembrane protein, is highly expressed on various types of cancer cells. By binding to its receptor PD-1 on T cells, PD-L1 significantly inhibits T cell activation and activity, thereby playing a crucial role in enabling tumor cells to evade immune surveillance (111). Antibodies that block PD-L1/PD-1 interactions have revolutionized cancer treatment, showing promising clinical outcomes in melanoma, lung, bladder, colorectal, and renal cell carcinoma. However, the response rate to PD-L1/PD-1 antibody therapy is less satisfactory in other cancers such as prostate, ovarian, and breast cancer. In breast cancer, PD-L1 has been found to be palmitoylated by zDHHC9 at the C272 site, enhancing its stability (33). Mutation of Cys272 to Ala dramatically abolishes mPD-L1 palmitoylation, or inhibiting the expression of zDHHC9 sensitizes breast cancer cells to T-cell killing, thereby repressing tumor growth (33). Another study discovered that S-palmitoylation of PD-L1 at Cys272 inhibits its ubiquitination-mediated lysosomal degradation. zDHHC3 is the primary acyltransferase responsible for PD-L1 S-palmitoylation, and inhibiting PD-L1 S-palmitoylation using 2-BP or silencing DHHC3 activates anti-tumor immunity in CRC (112). The same group further elucidated the regulatory role of S-palmitoylation in PD-1 stability in tumor cells. Mechanistically, zDHHC9 mediates PD-1 palmitoylation at C192, promoting its binding with recycling endosomes and preventing lysosome-dependent degradation (113). They demonstrated that palmitoylation of PD-1, but not PD-L1, promotes mTOR signaling and tumor cell proliferation.

S-palmitoylation of PD-L1 plays a crucial role in regulating its stability. It inhibits the monoubiquitination of PD-L1, thereby preventing its passage through the endosomal sorting complex required for transport (ESCRT). Consequently, the degradation of PD-L1 via the lysosomal pathway is blocked, leading to increased expression of PD-L1 on the cell surface. This increased expression of PD-L1 inhibits the cytotoxicity of T cells. In order to enhance the lethality of cytotoxic T cells against cancer cells, researchers developed a competitive peptide that specifically inhibits PD-L1, surpassing the commonly used universal palmitoylation inhibitor 2-BP in terms of specificity. As a result, the inhibitor reduced the expression of PD-L1 in tumor cells. These findings highlight the significant antitumor effects of targeted S-palmitoylation *in vitro* and offer promising opportunities for the development of new approaches in cancer immunotherapy.

## 5 Targeting protein S-palmitoylation for cancer treatment

Protein S-palmitoylation plays a crucial role in cancer progression and anti-tumor immunity, making it an attractive target for cancer therapy. Inhibitors of palmitoylation could be beneficial for anticancer treatment, as many oncogenes require S-palmitoylation modification for proper localization on the cellular membrane. Currently, there are no potent and specific inhibitors for zDHHC enzymes. While broad-spectrum protein S-palmitoylation inhibitors like 2-BP are commonly used to validate anticancer effects and have been employed in pre-clinical studies (39), they lack selectivity for individual zDHHC enzymes and can acylate other intracellular proteins, making them unsuitable as drug leads or therapeutic candidates (25). Through a FRET-based high throughput assay screening of over 350,000 compounds, two related tetrazole-containing compounds (TTZ-1 and TTZ-2) were identified that inhibit both zDHHC2 autopalmitoylation and substrate palmitoylation in cell-free systems (114). However, it remains to be investigated whether these compounds or their derivatives can inhibit other mammalian zDHHC enzymes in cancer or immune cells. Crystal structures of zDHHC20 and zDHHC15 have been reported (22, 114), and structure-guided methods can be used to develop inhibitors that target specific zDHHC enzymes at their binding domains (22). However, obtaining structures of additional zDHHC enzymes would be necessary to discover inhibitors that are specific to one enzyme over another. Moreover, since many proteins can be palmitoylated by multiple zDHHC enzymes, specific inhibitors for individual zDHHC enzymes may not be clinically useful as they may target multiple zDHHC family members. A potential solution to this challenge would be to develop inhibitors that can block the function of several specific palmitoylation enzymes for a single target.

Palmitate-derived palmitoyl-CoA supports protein palmitoylation in cancer cells or immune cells. Previous studies have shown that inhibiting palmitate generation by FASN inhibitors such as TVB-3166 or TVB-3664 significantly reduces palmitate generation and tubulin S-palmitoylation (115). Combining FASN inhibition with taxane treatment enhances the inhibition of *in vitro* tumor cell growth in lung, ovarian, prostate, and pancreatic cancers. Competitive inhibition of substrate S-palmitoylation is an effective method for targeting specific zDHHC enzymes. It is conceivable that a small molecule or polypeptide could inhibit zDHHC-protein substrate interactions, which are necessary for substrate S-palmitoylation. This competitive inhibition strategy could be used to develop more specific palmitoylation inhibitors. In a previous study, a polypeptide containing the PD-L1 (265aa-279aa) sequence, which includes the S-palmitoylation site of PD-L1, was developed (112). The polypeptide sequence near the S-palmitoylation site may inhibit the S-palmitoylation of endogenous PD-L1 by competitively binding to zDHHC3. The results show that this polypeptide can effectively reduce the S-palmitoylation of PD-L1, thereby significantly decreasing the expression of PD-L1 in tumor cells. This study suggests that a competitive inhibition strategy can effectively inhibit the S-palmitoylation of PD-L1.

Rather than inhibiting protein S-palmitoylation, it may sometimes be more effective to focus on preventing depalmitoylation. Several small molecule inhibitors have been developed for this purpose, including the pan-depalmitoylation inhibitor PalmB, as well as the APT1- and APT2-specific inhibitors ML348 and ML349 (116, 117). Inhibiting APT enzymes can suppress tumor formation by promoting the proper localization of SCRIB and enhancing the activity of melanocortin-1 receptor (MC1R) (118–120). In addition to regulating tumor suppressor function through inhibition of APT1/2, inhibiting lysosomal PPT1-mediated depalmitoylation may have a more direct toxic effect on cancer cells (121). Cells lacking PPT1 exhibit abnormal accumulation of palmitoylated protein in the ER (122). This protein leads to activation of the unfolded protein response (UPR) pathway and increased expression of chaperone proteins such as glucose-regulatory protein-78. The abnormal accumulation of protein also mediates caspase-12 and caspase-3 activation, ultimately inducing apoptosis (123). Inhibition of PPT1 by the natural product didemnin B may inhibit lysosomal acidification and induce apoptosis. In this case, apoptosis is caused by the loss of the rapidly degraded pro-survival protein MCL1. Apart from disrupting lysosome function, DQ661 or didemnin B might also cause protein synthesis inhibition, lysosomal membrane permeabilization, and apoptosis via mTOR signaling (124). DQ661 inhibits the mTOR pathway by preventing RAGC, MTOR, and other key molecules from associating with the lysosomal surface. PPT1 inhibition by HCQ or DC661 induces cGAS/STING/TBK1 pathway activation and interferon-β release in macrophages, enhancing the antitumor efficacy of anti-PD-1 Ab in melanoma (125).

## 6 Concluding remarks

S-palmitoylation plays a role in various aspects of tumor cell proliferation, metastasis (126), and apoptosis, impacting tumors by influencing antitumor immunity in the tumor microenvironment (127). Abnormal expression of zDHHCs, APTs, and PPTs, as well as changes in the S-palmitoylation status of cancer-associated proteins, have been observed in almost all types of cancer. Previous studies have shown that inhibition of some members of the zDHHC family such as zDHHC3, 5, 17, 9, 12 inhibited tumor progression and enhanced the therapeutic efficacy of PD-1/PD-L1 checkpoint antibody blockade in CRC, glioblastoma, leukemia, and pancreatic cancer (33, 80, 128, 129). These findings suggest that targeting either zDHHCs and PPTs or palmitoylated cancer-associated proteins could potentially benefit the treatment of different cancer types. However, it is important to note that inhibiting protein S-palmitoylation can have toxic effects on normal cells. Despite this caution, there is strong evidence supporting the targeting of PAT enzymes as a potential approach to treating cancer (12, 125). For instance, oncoproteins like RAS-family GTPases rely on S-palmitoylation to promote tumor formation. Inhibiting the function of oncoproteins by blocking this key post-translational modification is an appealing strategy, particularly for undruggable proteins such as RAS.

In recent years, there has been rapid development in tumor immunotherapy, which has shown promising results in some patients, offering the potential for long-lasting treatment. However, current cancer immunotherapy still faces challenges such as low response rates and the risk of serious immune-related adverse events. The effectiveness of tumor immunotherapy largely depends on the tumor microenvironment, particularly the tumor immune microenvironment. Therefore, understanding the regulatory mechanism of S-palmitoylation in the tumor microenvironment can serve as a theoretical foundation for intervening in S-palmitoylation to regulate the tumor immune microenvironment. This understanding can pave the way for improving the efficacy of immunotherapy by providing a new direction.

In summary, the investigation of the regulatory mechanism of protein S-palmitoylation, a post-translational modification, on the tumor immune microenvironment is a crucial and promising research area in the field of tumor immunity. Understanding this mechanism can have significant clinical implications and contribute to the advancement of tumor immunotherapy.

## Author contributions

YC: Writing – original draft, Writing – review & editing. YL: Conceptualization, Data curation, Project administration, Resources, Supervision, Writing – original draft, Writing – review & editing. LW: Conceptualization, Data curation, Investigation, Project administration, Resources, Supervision, Writing – original draft, Writing – review & editing.
